# A comparison between different ways to assess demands-abilities fit in higher education: Empirical results and recommendations for research practice

**DOI:** 10.3389/fpsyg.2022.896710

**Published:** 2022-07-22

**Authors:** Carla Bohndick, Jonas Breetzke, Tom Rosman

**Affiliations:** ^1^Hamburg Center for University Teaching and Learning (HUL), University of Hamburg, Hamburg, Germany; ^2^Research Literacy Unit, Leibniz-Institute for Psychology (ZPID), Trier, Germany

**Keywords:** person-environment fit theory, demands-abilities fit, measurement approaches, response surface analysis, difference score, study success, grades, study satisfaction

## Abstract

Researchers studying person-environment fit can choose between various measurement approaches. Even though these measures are distinctly different, they often get used interchangeably, which makes interpreting the results of person-environment fit studies difficult. In the present article, we contrast the most commonly used measurement approaches for person-environment fit in higher education and compare them in terms of explained variance. We obtained data on the fit as well as subjective and objective study-related outcomes of *N* = 595 university students. We analyzed the fit between the demands of the study program and the abilities of the student, using the algebraic, squared and absolute difference score, response surface analysis (RSA), and direct fit as measurement approaches. Our results indicate that RSA explains the most variance for objective outcomes, and that direct fit explains the most variance for subjective outcomes. We hope that this contribution will help researchers distinguish the different measurement approaches of demands-abilities fit (and ultimately person-environment fit) and use them accordingly.

## Introduction

Person-environment fit (P-E fit) theory (Edwards et al., 2006) suggests that a fit between personal factors (e.g., learning skills) and environmental factors (e.g., learning demands) leads to positive outcomes, for example, study performance or satisfaction. When assessing fit, researchers can choose between many distinctly different measurement approaches that vary in their underlying assumptions (Edwards, 1993; Bohndick et al., 2018). Accordingly, there is only a low to moderate correlation between different approaches, and research suggests that some assess different theoretical constructs altogether (Edwards et al., 2006). However, the concept of P-E fit is often misunderstood (Kristof-Brown and Billsberry, 2013), and even though measurement approaches are one of the most important moderators of fit effects (Kristof-Brown et al., 2005) they often get used interchangeably (Cable and DeRue, 2002). It is therefore striking that, to our knowledge, no studies have empirically compared the different measurement approaches for P-E fit in terms of their explained variance. Such a comparison would help to distinguish the various measurement approaches and identify how they influence different outcomes.

The present study contrasts the most commonly used measurement approaches for P-E fit and compares them in terms of their explained variance. We focus on P-E fit in higher education, a context with many applications of P-E fit theory in recent years (Li et al., 2013; Etzel and Nagy, 2016; Bohndick et al., 2018; Rocconi et al., 2020), and analyze the fit between the abilities of university students and the demands of their study programs (demands-abilities fit). Our goal thereby is to give recommendations on which measurement approach should be used in different scenarios.

### Measurement approaches

Most measurement approaches can be put into one of two dominant research streams: (1) The indirect assessment of fit as the interaction between personal and environmental factors, or (2) the direct assessment of fit as an internal feeling (Kristof-Brown and Billsberry, 2013). When fit is assessed indirectly, researchers separately assess the person as well as the environment and compare both factors *ex post* (Bohndick et al., 2018). This is commonly done by either collapsing a pair of component measures into a congruence index (Edwards, 1994) or by calculating a polynomial regression model and evaluating it using Response Surface Analysis (RSA) (Humberg et al., 2019). We will describe these measurement approaches and their underlying assumptions in the following section.

#### Difference scores

Numerous studies have used difference scores to collapse person and environment variables into a single index (Edwards, 1994), that represents the overall level of fit (Edwards, 1993). The most common indexes are the algebraic (*X-Y*), squared (*X-Y*)^2^, and absolute | *X-Y*| difference score (Edwards, 1994). The algebraic difference score assumes a linear relationship between fit and the outcome (Bohndick et al., 2018). Fit is understood as one factor exceeding the other (Bohndick et al., 2018), and a higher score indicates a better fit. The score assigns equal weight to differences of higher magnitude but distinguishes between positive and negative differences (Edwards, 1993). Squared and absolute difference scores consider fit as the perfect congruence between person and environment (Bohndick et al., 2018). A perfect fit results in a difference score of zero, with higher scores indicating a higher misfit. The squared difference score assumes an inversely U-shaped relationship between fit and the outcome (Bohndick et al., 2018). It assigns greater weight to differences of higher magnitude and does not differentiate between positive and negative differences (Edwards, 1993). Absolute difference scores assume an inversely V-shaped relationship (Bohndick et al., 2018), assigning equal weight to differences of increasing magnitude, otherwise follow the same logic as squared difference scores (Edwards, 1993).

Even though difference scores have been used for decades, a lot of problems surround their use, which is why they have been extensively criticized in the literature (Edwards, 1993). For example, they have been accused of discarding information and being overly restrictive. Furthermore, it has been claimed that their use introduces methodical problems concerning conceptual clarity and that they are insensitive to the sources of the differences (Edwards, 1993).

#### Response surface analysis

In recent years, RSA gained a lot of attention as an alternative to difference scores (Humberg et al., 2019). RSA estimates a polynomial regression model that includes both person and environment as well as their higher order terms as predictor variables (Kristof-Brown et al., 2005). This enables researchers to detect and interpret fit patterns using a three dimensional graph of the model and several surface parameters as guidelines (Humberg et al., 2019). As a result, RSA is able to depict more complex relationships, and allows for more elaborate tests while removing a lot of the shortcomings of difference scores (Edwards, 1994).

However, RSA comes with a few problems, too, as it requires at least two times as many participants as linear main effects (Humberg et al., 2019). Using RSA can be quite complex, and the interpretation might be challenging for someone not accustomed to the procedure (Humberg et al., 2019). In addition, there are no possibilities to collapse different dimensions into one score, which complicates analyses of complex fit hypotheses. Additionally, and in contrast to the other approaches, RSA does not provide researchers with a fit score that can be used for further calculations (Edwards, 1995). A fit score, however, might be necessary for example when researchers are interested in the predictors of fit—an important topic for the development of P-E fit theory and its application.

#### Direct fit

When measuring fit directly, individuals report their perceived fit to researchers (Edwards et al., 2006), for example by rating statements such as “The match is very good between the demands of my schoolwork and my personal ability” (Li et al., 2013, p. 168). While indirect measures weigh person and environment equally, direct fit lets respondents apply their own weighting scheme (Kristof-Brown et al., 2005). This leads to a greater amount of cognitive processing in the respondents (Kristof-Brown et al., 2005) and removes the need for researchers to choose a specific combination rule (Bohndick et al., 2018).

The direct fit does not share the methodical problems of difference scores resulting from collapsing two variables into a single index. Instead, when using direct fit, the comparison process of person and environment is all done in the respondent’s mind, and information about the independent effects of person and environment is lost (Kristof, 1996). In addition, the direct measure of fit is susceptible to common method bias (Kristof-Brown et al., 2005).

### Comparing the different measurement approaches

No measurement approach can do it all, and there are arguments for and against the use of every previously mentioned approach. There has been a long discussion on whether the direct or the indirect measures are better suited to assess P-E fit, with both sides holding valid arguments (Kristof-Brown and Billsberry, 2013). Speaking in favor of direct measurement, a number of studies suggest that the direct approach leads to larger effect sizes for a majority of outcomes, which is why the direct fit might be the better predictor of outcomes (Verquer, 2002; Kristof-Brown et al., 2005) and closer to human decision making (Kristof-Brown and Billsberry, 2013). However, direct fit is strongly influenced by affect (Kristof-Brown and Billsberry, 2013), susceptible to common method bias (Kristof-Brown et al., 2005), closely resembles an attitude (Kristof-Brown and Billsberry, 2013) and carries additional meaning beyond the perceived person and environment (Edwards et al., 2006)—resulting in considerable criticism. But seeing the direct and the indirect measurement approaches as competitors might hold less value, as studies find little correlation between both approaches and indicate that they assess different theoretical constructs (Bohndick et al., under review; Edwards et al., 2006). In line with this idea, Kristof (1996) suggests that the indirect measurement as the actual match between person and environment should have a stronger effect on process or performance outcomes, while the direct measure—as the feeling of fit—closely resembles an attitude and should therefore have a stronger effect on individual attitudinal outcomes.

Most of the aforementioned studies, however, do not consider RSA, which has only recently been established as an alternative to difference scores (Humberg et al., 2019). This is striking since a particularly strong association between RSA and outcomes can be expected based on the results of a qualitative review by Kristof-Brown et al. (2005), which suggests that polynomial regression models produce larger correlations compared to other types of fit measures. This may be because polynomial regression models include main and quadratic effects not captured by direct measures or difference scores (Kristof-Brown et al., 2005). In addition, RSA is suited to depict more complex relationships than other measures (Kristof-Brown et al., 2005), thus allowing for a more precise comparison and the testing of more complex hypotheses (Kristof-Brown et al., 2005).

### The present study

All in all, research comparing the different measurement approaches is mostly theoretical and empirical research has yet failed to distinguish the approaches and how they influence different outcomes. To address this, this study aims to contrast the most commonly used measurement approaches for P-E fit and give recommendations on which approach should be used in different circumstances. One possible way to do so is to compare them in terms of their explained variance for different outcome variables. This results in the following research question: Which of the commonly used measurement approaches for P-E fit explain the most variance in terms of *R*^2^ in different subjective and objective study-related outcomes?

To address our research question, we empirically assess the fit between the demands of the study program and the students’ abilities. As subjective study-related outcomes, we concentrate on study satisfaction and intent to leave, and we use grades as the most suitable proxy for the objective study performance. Based on previous research outlined above, we posit three hypotheses on how the different measurement approaches differentially predict study-related outcomes in terms of explained variance.

*Hypothesis 1: For both subjective and objective study-related outcomes, we expect RSA to explain the most variance.*

Polynomial regression models include main and quadratic effects and thus allow to depict more complex relationships than other measures (Kristof-Brown et al., 2005), which in turn should increase the amount of explained variance. Empirically, this is supported by Kristof-Brown et al.’s (2005) review, which suggests that polynomial models produce larger correlations than other types of fit measures (Kristof-Brown et al., 2005).

*Hypothesis 2: For subjective study-related outcomes, we expect the direct measurement approach to explain the second-largest share of the variance.*

The direct and indirect measurement approaches assess different theoretical constructs that have yet to be separated from one another (Bohndick et al., under review; Edwards et al., 2006). In line with these findings, researchers suggest that the direct approach, as the feeling of fit, closely resembles an attitude and should therefore have a strong effect on other attitudinal outcomes such as study satisfaction and intent to leave (Kristof, 1996; Kristof-Brown and Billsberry, 2013).

*Hypothesis 3: For objective study-related outcomes, we expect the absolute difference score to explain the second largest share of the variance.*

The indirect approach as the actual match between person and environment is theorized to better predict process and performance outcomes (Kristof, 1996; Kristof-Brown and Billsberry, 2013). We, therefore, expect difference scores to explain more variance in objective outcomes than the direct measure. In line with existing research on P-E fit, we assume that the congruence between abilities and demands is more beneficial compared to abilities exceeding the demands (Kristof-Brown et al., 2005; Bohndick et al., 2018). As a result, we expect the relationship between demands–abilities fit and objective outcomes to be either inversely V-shaped (absolute difference) or inversely U-shaped (squared difference). Furthermore, previous research suggests that the absolute score explains slightly more variance in academic success compared to the squared and algebraic difference (Bohndick et al., 2018).

## Methods

### Sample and procedure

We collected data of *N* = 595 university students from German-speaking countries using an online questionnaire. Participants were recruited primarily in Facebook groups and mailing lists until there was no longer any significant growth in participation rates. As an incentive, participants could take part in a lottery of 10 Amazon vouchers worth 50 Euros each. To ensure that the lottery does not increase invalid participation (e.g., participation by individuals not studying at a university, multiple participation to increase one’s chances of winning the lottery), only students with a university e-mail address were eligible for the lottery. Participants were from heterogeneous study disciplines including teacher education, economics, psychology, mathematics, medicine, and sociology. Mean age was *M* = 22.89 years (*SD* = 3.53). The majority of the participants were female (*n* = 475; 79.57%). On average, students were in their 5.21th semester (*SD* = 4.00).

The data contained only few missing values, mostly for grades (*k* = 132). For the RSA, missing values were dealt with using Full Information Maximum Likelihood estimates. For the correlation tests, missing values were omitted using pairwise deletions.

### Measures

To assess fit indirectly, students were asked to separately rate, (a) the ability level required for their studies, and (b) their own ability level on a five-point Likert scale ranging from “very low” (1) to “very high” (5; see Table 1 for details). For direct fit, we used four items based on studies with direct fit as measurement (e.g., Li et al., 2013). These items’ response format was a five-point Likert scale ranging from “does not apply at all” (1) to “fully applies” (5). Study success was assessed with regard to two subjective criteria—study satisfaction (following Westermann et al., 1996) and intent to leave (following Blüthmann, 2014)—and one objective criterion where students were asked to specify their last four study grades.

**TABLE 1:** Measurement instruments.

Construct	*k*	Items	*M* (*SD*)	Q1	*Md*	Q3	α
Demands	3	General study ability; general ability to learn; general academic ability	4.09 (0.67)	3.67	4.00	4.67	0.82
Abilities	3	General study ability; general ability to learn; general academic ability	3.81 (0.65)	3.33	4.00	4.00	0.79
Direct fit	4	The fit between my personal skills and the requirements of my studies is very good.	3.80 (0.74)	3.25	4.00	4.25	0.89
Study satisfaction	3	I enjoy what I study; I am satisfied with my current choice of studies; I find my studies interesting	4.11 (0.78)	3.67	4.33	4.67	0.85
Intent to leave	3	If I had a good alternative, I would quit my studies; I have considered to drop out; If things keep going the way they are, I’m pretty sure I’ll quit my studies	1.77 (0.96)	1.00	1.33	2.33	0.76
Grades	4	Mean of the last four grades	2.07 (0.69)	1.50	2.00	2.50	0.65

k, number of items per scale; M, Mean; SD, Standard Deviation; Q1, Lower quartile; Md, Median; Q3, Upper quartile; α, Cronbach’s alpha. All items were administered in German language.

Reliability analyses yielded acceptable to very good values for Cronbach’s alpha (between α = 0.65 and α = 0.89). Individual items and their descriptive statistics can be found in Table 1.

### Statistical analysis

All calculations were done in R (version 4.1.2; R Core Team, 2021). Difference scores were created by subtracting the demands of the study program from the abilities of a person (algebraic difference), and squaring (squared difference), or taking the absolute value (absolute difference) of each score. To calculate the explained variance in terms of *R*^2^ (and adjusted *R*^2^ for RSA), the fit scores of the algebraic, squared and absolute difference scores were then correlated with study satisfaction, intent to leave, and grades.

For the RSA, the package RSA (version 0.10.4; Schönbrodt and Humberg, 2021) was used. As suggested by Humberg et al. (2019), we standardized the two predictor variables X and Y. Subsequently, we calculated the full polynomial model as well as seven constrained nested models, and used the best model according to the Akaike Information Criterion (AIC) (see Schönbrodt, 2016).

## Results

Table 2 reports the explained variance in terms of *R*^2^ and the direction of the correlation of each measurement approach for the outcomes study satisfaction, intent to leave, and grades. For the RSA, the direction of the correlation is not indicated, as the models can indicate simple main effects as well as complex fit relations.

**TABLE 2:** Explained variance of each measurement approach for different outcomes.

	Difference scores			RSA	Direct fit
	Squared	Absolute	Algebraic		
**Study satisfaction**	0.064 (–)	0.058 (–)	0.037 (+)	0.142	0.251 (+)
**Intent to leave**	0.099 (+)	0.083 (+)	0.072 (–)	0.150	0.164 (–)
**Grades**	0.018 (+)	0.020 (+)	0.051 (–)	0.097	0.055 (–)

Columns show the explained variance in terms of R^2^ (and adjusted R^2^ for RSA) as well as the direction of the effect (in parentheses); (+) indicates a positive, (–) indicates a negative correlation with the respective outcome.

The results indicate that, in general, a greater demands-abilities fit is associated with higher study satisfaction, a lower intent to leave and better grades. Out of the five measurement approaches, RSA explains the most variance for grades and the direct fit explains the most variance for both subjective study-related outcomes. Difference scores explain significantly less variance than RSA and direct fit. The only exception are grades: Here, the algebraic difference score explains almost as much variance as the direct fit, which nevertheless is only half of the amount of explained variance by RSA.

## Discussion

The present study contrasts the most commonly used approaches to measure P-E fit and evaluates them in terms of their explained variance for both subjective and objective study-related outcomes. We measure demands-abilities fit of *N* = 595 university students, and estimate the amount of explained variance using five different measurement approaches. Our results indicate that the explained variance of the models is highly dependent on the measurement approach and ranges from 2 to 25%, which further underlines the importance of taking the measurement approach into account when designing or interpreting a study on P-E fit.

Nevertheless, none of the three hypotheses were fully supported. Instead, the following picture emerges: Direct fit explains the most variance for subjective study success criteria and RSA explains the most variance for objective study criteria. Difference scores always explain less variance than one of the other two. On the one hand, this is surprising, given the fact that existing research indicates that RSA is one of the most powerful approaches for analyzing P-E fit (Edwards, 1994; Humberg et al., 2019). On the other hand, our results support the notion of prior research that the direct fit has a stronger influence on attitudes such as satisfaction and intent to leave (Kristof-Brown et al., 2005).

When interpreting our results, a few limitations have to be considered. First, our results and recommendations are limited to the higher education context, and do not allow us to draw direct conclusions for other research areas. However, as we specifically chose outcomes (satisfaction, intent to quit and performance) that are frequently used in the organizational context, we consider it likely that our results are transferable. Second, we focus on demands-abilities fit to the exclusion of other types of fit (e.g., needs-supply fit). Demands-abilities fit is frequently studied in the higher education context (Li et al., 2013; Etzel and Nagy, 2016; Bohndick et al., 2018) and provides a good starting point for our analysis. Nevertheless, additional research needs to examine whether our results are transferrable to different types of fit. Third, all fit measures were assessed using self-reports. As a result, our measures relate to the subjective perception of the participants instead of assessing objective traits or characteristics. Further studies might, for example, use actual test results to assess students’ abilities and thereby incorporate objective fit into their analysis. Fourth, we used a cross-sectional design and collected our data at one point in time. This is necessary to ensure that each measure refers to the same experience and can be compared (Edwards et al., 2006), but inhibits causal conclusions. A longitudinal design would enable a better approximation of the underlying causal effect. Finally, we focused on explained variance to compare the different approaches, which could have influenced our results and recommendations. Especially RSA could be the beneficiary here, as the model is optimized regarding model select criteria (AIC and adj. *R*^2^). Comparing the measurement approaches in terms of additional criteria, such as reliability and practicality, could be a worthwhile project and further enhance our understanding of the different measurement approaches.

Despite these limitations, our study can contribute to the literature on P-E fit by distinguishing the commonly used measurement approaches and by giving insights on how they influence different outcomes. These results can help researchers interpret studies using the different measurement approaches.

## Data availability statement

The raw data supporting the conclusions of this article will be made available by the authors, without undue reservation.

## Ethics statement

Ethical review and approval was not required for the study on human participants in accordance with the local legislation and institutional requirements. The patients/participants provided their written informed consent to participate in this study.

## Author contributions

CB and TR conceived and planned the study. TR conducted the data collection, reviewed and revised the manuscript, and prepared it for publication. CB and JB analyzed the data and prepared a first draft of the manuscript. All authors contributed to the article and approved the submitted version.

## Conflict of interest

The authors declare that the research was conducted in the absence of any commercial or financial relationships that could be construed as a potential conflict of interest.

## Publisher’s note

All claims expressed in this article are solely those of the authors and do not necessarily represent those of their affiliated organizations, or those of the publisher, the editors and the reviewers. Any product that may be evaluated in this article, or claim that may be made by its manufacturer, is not guaranteed or endorsed by the publisher.
